# Metabolic changes of the acetogen *Clostridium* sp. AWRP through adaptation to acetate challenge

**DOI:** 10.3389/fmicb.2022.982442

**Published:** 2022-12-07

**Authors:** Soo Jae Kwon, Joungmin Lee, Hyun Sook Lee

**Affiliations:** ^1^Marine Biotechnology Research Center, Korea Institute of Ocean Science and Technology, Busan, South Korea; ^2^Department of Marine Biotechnology, University of Science and Technology, Daejeon, South Korea

**Keywords:** acetogen, *Clostridium* sp. AWRP, adaptive laboratory evolution, transcriptome, acetate stress

## Abstract

In this study, we report the phenotypic changes that occurred in the acetogenic bacterium *Clostridium* sp. AWRP as a result of an adaptive laboratory evolution (ALE) under the acetate challenge. Acetate-adapted strain 46 T-a displayed acetate tolerance to acetate up to 10 g L^−1^ and increased ethanol production in small-scale cultures. The adapted strain showed a higher cell density than AWRP even without exogenous acetate supplementation. 46 T-a was shown to have reduced gas consumption rate and metabolite production. It was intriguing to note that 46 T-a, unlike AWRP, continued to consume H_2_ at low CO_2_ levels. Genome sequencing revealed that the adapted strain harbored three point mutations in the genes encoding an electron-bifurcating hydrogenase (Hyt) crucial for autotrophic growth in CO_2_ + H_2_, in addition to one in the *dnaK* gene. Transcriptome analysis revealed that most genes involved in the CO_2_-fixation Wood-Ljungdahl pathway and auxiliary pathways for energy conservation (e.g., Rnf complex, Nfn, etc.) were significantly down-regulated in 46 T-a. Several metabolic pathways involved in dissimilation of nucleosides and carbohydrates were significantly up-regulated in 46 T-a, indicating that 46 T-a evolved to utilize organic substrates rather than CO_2_ + H_2_. Further investigation into degeneration in carbon fixation of the acetate-adapted strain will provide practical implications for CO_2_ + H_2_ fermentation using acetogenic bacteria for long-term continuous fermentation.

## Introduction

Since the autotrophic production of acetate from CO_2_ and H_2_ was first discovered in *Clostridium aceticum*, a number of acetogenic bacteria, including *Moorella thermoacetica* (formerly *Clostridium thermoaceticum*), *Acetobacterium woodii*, and *Thermoanaerobacter kivui*, have been studied for their biotechnological potential for CO_2_ reduction (Wieringa, 1939; Groher and Weuster-Botz, 2016; Hoffmeister et al., 2016; Hu et al., 2016; Basen et al., 2018). These acetogens assimilate CO_2_ or CO *via* the reductive acetyl-CoA pathway, also known as the Wood-Ljungdahl (WL) pathway. Some acetogens, such as *Clostridium ljungdahlii* and *Clostridium autoethanogenum*, are of great interest for their ability to autotrophically produce ethanol and 2,3-butanediol, and have a wide range of applications for bioconversion of waste gases containing significant amounts of CO (Bengelsdorf et al., 2018). Due to the flexibility of the gas utilization of the WL pathway, these acetogens have recently attracted increased interest in the direct utilization of CO_2_ as well as the utilization of CO-containing gases to address concerns about climate change (Jin et al., 2021; Liew et al., 2022). In general, growth in CO_2_ + H_2_ yields lower cell densities than CO possibly because the redox potential of H_2_ is higher than that of CO (Schuchmann and Müller, 2014; Mock et al., 2015). Although fermentation profiles may vary depending on growth conditions and species, acetate is often the main fermentation product (Heffernan et al., 2020; Zhu et al., 2020). However, little is known about the fermentation kinetics and physiology of these alcohol-producing acetogens grown on CO_2_ + H_2_.

Acetic acid, the major fermentative compound of acetogen, is a toxic compound for a variety of microorganisms (Trček et al., 2015). The effects of acetate have been well studied in *Escherichia coli*, in which acetate accumulation is often observed as a result of overflow metabolism and reduces the performances of biotechnological processes employing this bacterium (Kleman and Strohl, 1994; Lee, 1996). Acetic acid is a weak acid with a p*K*_a_ of 4.75, and when the culture pH is low, undissociated acids can easily penetrate the cytoplasmic membrane. Therefore, acetate accumulation can lead to cytoplasmic acidification and disruption of the transmembrane pH gradient, particularly adversely affecting the growth of anaerobes with lower ATP yields than aerobes by increasing cellular maintenance costs (Valgepea et al., 2017a).

Adaptive laboratory evolution (ALE) has been implemented in a number of studies to gain insight into the genetic identity that underlies phenotypes (Goodarzi et al., 2010; Long and Antoniewicz, 2018). ALE has also been employed to improve the tolerance of production strains to products or inhibitory compounds present in the raw material (Fernandez-Sandoval et al., 2012; Wallace-Salinas and Gorwa-Grauslund, 2013; Ju et al., 2016). So far, only a few studies have been reported on acetogens, focusing on improving the utilization of C_1_ substrates, especially CO and methanol, which are known to cause severe substrate inhibition in these bacteria (Tremblay et al., 2015; Kang et al., 2020). It has been reported that the acetate tolerance of two *Moorella* species could be successfully improved through iteration of random mutagenesis and selection performed using glucose as the major carbon source (Reed et al., 1987).

In our previous study, *Clostridium* sp. AWRP (hereinafter referred to as AWRP), a novel ethanol-producing acetogen isolated from wetland soil in Ansan, Republic of Korea, displayed high ethanol yields when grown in CO-containing gases (Lee et al., 2019). As other acetogenic bacteria, this organism can also use CO_2_ and H_2_, producing acetate and ethanol. In this study, we investigated how this bacterium would respond to acetate stress during autotrophic growth using CO_2_ and H_2_.

## Materials and methods

### Culture media

LBFA medium, which was used for propagation of the wild-type AWRP, contained the following ingredients: D-fructose (Junsei Chemical, Tokyo, Japan), 5 g L^−1^; Bacto™ yeast extract (BD Biosciences, CA), 5 g L^−1^; Bacto™ tryptone (BD), 10 g L^−1^; NaCl (Duchefa Biochemie, Haarlem, Netherlands), 0.5 g L^−1^; CH_3_COONa∙3H_2_O (Junsei), 5 g L^−1^; and L-cysteine hydrochloride (Merck Korea, Seoul, South Korea), 0.5 g L^−1^. RM medium was used for autotrophic cultivation of AWRP and its derivatives, with minor modifications from the previous study (Lee et al., 2019). Briefly, the medium was supplemented with Bacto™ yeast extract (at different concentrations depending on experiments) and 20 mg L^−1^ L-methionine (a potential auxotrophic nutrient in AWRP). When necessary, ammonium acetate was supplemented to the medium at 5 g L^−1^ (described as total acetate concentration throughout the paper; equivalent to 83 mM) or 10 g L^−1^ (167 mM). The concentrate ammonium acetate stock was prepared by adjusting the pH of 100 g L^−1^ acetic acid solution at 5.0 with concentrate ammonia solution.

### Adaptive laboratory evolution under ammonium acetate challenge

Adaptive laboratory evolution (ALE) was performed using wild-type AWRP as the parent strain. The culture was conducted according to the previously described procedure (Lee et al., 2019; Kwon et al., 2022). Cells were grown in 125-mL serum bottles filled with 20 mL of modified RM medium supplemented with 0.5 g L^−1^ yeast extract and 5 g L^−1^ ammonium acetate. A gas mixture of 20% CO_2_ and 80% H_2_ was used as the growth substrate. Once the cells consumed 60 to 70% of CO_2_ present in the headspace, which corresponds nearly to the mid-to-late exponential phase, a 10% (v/v) inoculum was transferred to 20 mL of fresh medium. All the cultures were performed at 37°C, 180 RPM of agitation. To isolate acetate-adapted colonies, the mid-exponential culture after the 46^th^ transfer was spread on RM agar containing 5 g L^−1^ ammonium acetate with serial dilutions, and was grown in a pressure-resistant container charged with the gas mixture at 100 kPa. One isolate, designated as 46 T-a, was chosen from 8 randomly picked colonies for further investigation.

### Small-scale cultivation in serum bottles

For seed cultures, strains were grown in 20 mL of the RM medium supplemented with 0.5 g L^−1^ yeast extract and 20 mg L^−1^ L-methionine contained in a 125-mL serum bottle. The headspace was charged with CO_2_ + H_2_ at 100 kPa (gauge pressure). For wild-type AWRP, a 5% of an LBFA culture was used as an inoculum for seed culture. In the case of 46 T-a, the seed culture was grown by inoculating a frozen stock prepared from an autotrophic culture because this strain grows slowly in LBFA medium. The main culture was performed under the same condition, starting with inoculation of 10% of the actively growing seed culture (OD_600_ ~ 0.5). All cultures growing in CO_2_ + H_2_ were conducted at 37°C, 180 RPM agitation on a rotary shaker.

### Cultivation in serum bottles with gas recharge

The procedures and culture conditions were same as those for the small-scale cultures, except that the main cultures were carried out using 1-L serum bottles (Chemglass Life Sciences, Vineland, NJ, United States) containing 100 mL of the RM medium. When the residual CO_2_ levels in the headspace dropped below 20% of the initial partial pressure, the headspace was replaced and finally pressurized to 100 kPa with a fresh gas mixture.

### Bioreactor experiments with continuous gas supply

The detailed configuration and operating procedures of the bioreactor have already been described elsewhere with some modifications (Lee et al., 2019). RM medium supplemented with 2.0 g L^−1^ yeast extract and 20 mg L^−1^ L-methionine and was used for both seed and main cultures to replenish more organic carbon sources for batch cultures. Seed cultures were grown in 1-L bottles with 160 mL of RM medium. Main cultures were conducted by transferring 160 mL of a seed culture grown to OD_600_ ~ 0.5 in a 1-L serum bottle, into a bioreactor containing 1.44 l of RM medium. The bioreactor was operated at 37°C with 500 rpm of agitation, and the pH was controlled at 5.0 by addition of 7.5 N NH_4_OH. Substrate gases (20% CO_2_ and 80% H_2_) were continuously fed into the bioreactor at a flow rate of 0.05 vvm without pressurization.

### Resting cell assay

AWRP and 46 T-a were grown autotrophically in 1-L serum bottles containing 100 mL RM medium supplemented with 0.5 g L^−1^ of yeast extract and 20 mg L^−1^ L-methionine. RM medium without supplementation of L-methionine and yeast extract was used as a buffer for resting cell assay. For AWRP, cells at mid-exponential phase (OD_600_ ~ 0.25) were harvested from 160 mL of culture broth (2 bottles per assay) by centrifugation at 4000 × g for 10 min at room temperature. The harvested cells were washed once with 20 mL of buffer and finally resuspended in 20 mL of buffer. The cell resuspension was transferred to a 160-mL serum bottle and incubated at 37°C, 180 RPM. The headspace was charged to 100 kPa with a CO_2_ + H_2_ gas mixture prior to incubation. For 46 T-a, resting cell assay was performed with the same procedure, except that 80 mL of mid-exponential culture (OD_600_ ~ 0.5) was used per assay.

### Analytical methods

Cell growth was determined by measuring the optical density at 600 nm (OD_600_) with a UV–Visible spectrophotometer (Biophotometer Plus; Eppendorf, Hamburg, Germany). Headspace gas composition was determined using a gas chromatograph (YL 6100; YL Instrument Co., Anyang, Republic of Korea) equipped with Porapak N (45/60 mesh, 10 ft., × 1/8 in., Supelco) and 13X molecular sieve (3 ft. × 1/8 in., Supelco) column. Analyses were performed using 100 μl injection with the inlet temperature of 150°C. Argon was used as the carrier gas at a flow rate of 30 mL min^−1^. The oven was maintained at 40°C. The temperatures of the thermal conductivity detector (TCD) and the flame ionization detector (FID) were 150 and 250°C, respectively. The concentrations of metabolites in the culture broth were determined using HPLC-RID system (YL 9100; YL Instrument Co.) equipped with Rezex™ ROA (300 × 7.8 mm, Phenomenex Inc., CA) column with aqueous sulfuric acid solution (2.5 mM) as the mobile phase. The column temperature and the flow rate of mobile phase were 60°C and 0.6 mL min^−1^, respectively.

### Whole genome sequencing and identification of mutations

Genomic DNA of 46 T-a was extracted using a Genomic-tip 20/G (Qiagen, Düsseldorf, Germany) according to the manufacturer’s protocol. Genome sequencing was performed at DNALINK, Inc. (Seoul, South Korea). Libraries for sequencing were constructed using the Nano DNA Library Prep Kit (Illumina, United States) and sequenced on a NovaSeq 6,000 system (Illumina). The BCL files were converted into FASTQ files and demultiplexed with Bcl2fastq v2.20 (Illumina). Quality controls of the raw data were performed with FastQC v0.11.2. The high-quality reads were mapped onto the reference genome of AWRP (NCBI accession No. CP029758) with bwa v0.7.12-r1039 (Li and Durbin, 2009; McKenna et al., 2010). The resulting BAM files were re-aligned with the IndelRealigner tool implemented in Genome Analysis Toolkit (GATK) v3.5-0-g36282e4 (Van der Auwera and O'Connor, 2020), and the base quality scores were re-calibrated with the GATK Base Quality Score Recalibration tool. Variant call was performed with GATK UnifiedGenotyper. All variant calls were verified by Sanger sequencing using the primers shown in Supplementary Table S1.

### RNA isolation

**Figure 1:**
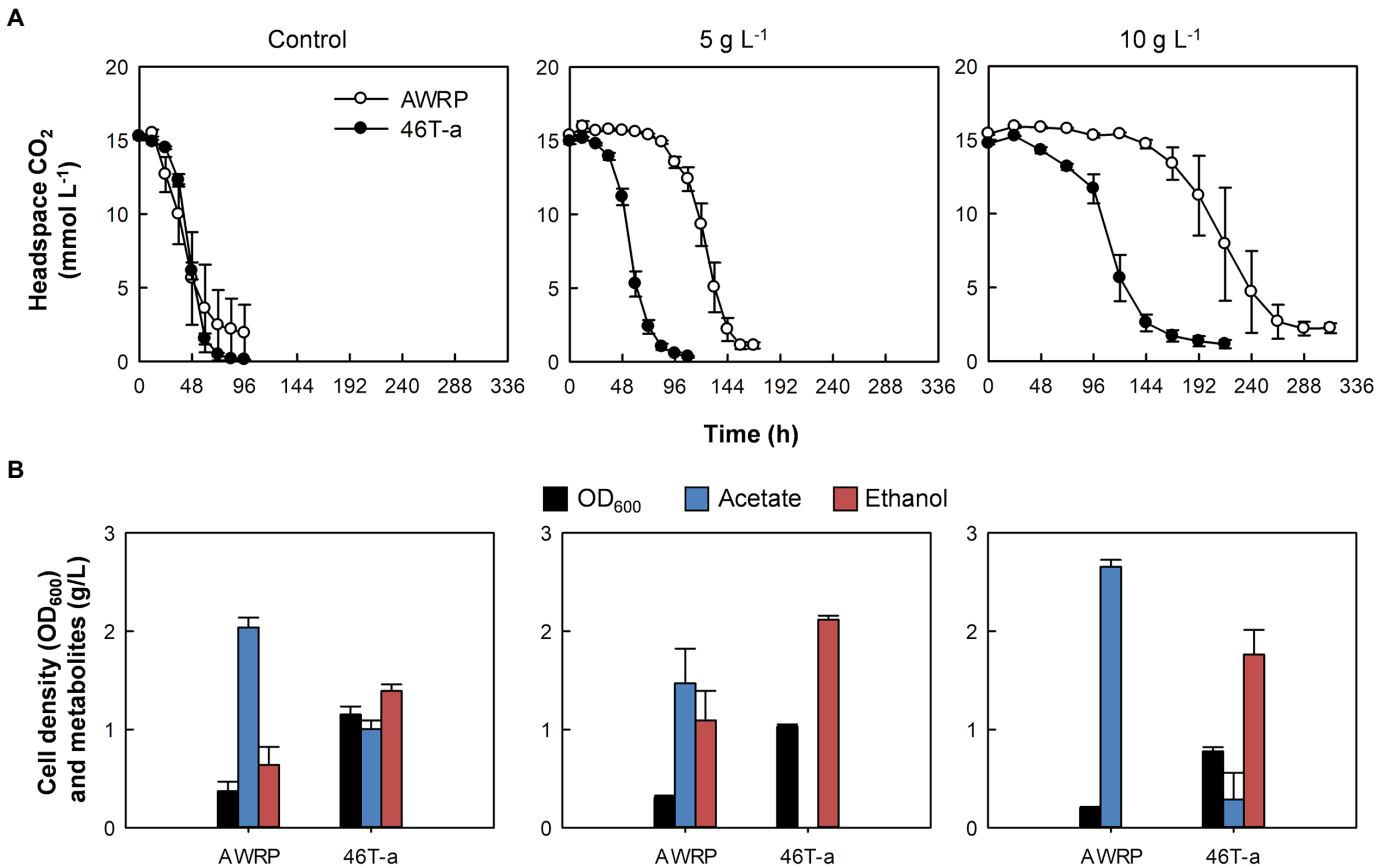
Comparison of wild-type AWRP and 46 T-a in the absence or presence of exogenous acetate. **(A)** Headspace CO_2_. **(B)** Cell mass and metabolites produced. Samples were taken with the last sampling of CO_2_ (see panel **A**). Acetate titers were expressed as net values obtained by subtracting the initial value from the final value for each bottle. Cultivation of each strain was conducted in triplicate and average values are shown. Error bars represent one standard deviation.

To isolate total RNA, an approximately equal number of the cells (4 [OD_600_ × mL]) (e.g., 4 mL culture was collected when OD_600_ = 1) were collected from the bioreactor at the exponential and the stationary phase (see Figure 1 for details of the sampling points). To preserve RNA integrity and transcriptome profile, the collected samples were immediately mixed with 2 volume of RNAProtect™ Bacteria reagent (Qiagen) and then incubated at room temperature for 20 min. After being harvested at 5000 × g for 10 min, cells were resuspended in 100 μl of lysozyme solution (15 mg mL^−1^ in 30 mM Tris∙Cl, 1 mM EDTA, pH 8.0), with the addition of 10 μl of proteinase K solution (Qiagen). The cells were incubated for 20 min at room temperature with periodic tapping every 3 min. 1 mL of RiboEX solution (GeneAll, Seoul, South Korea) was mixed with the enzyme-treated cells to extract total RNA according to the manufacturer’s protocol.

### Transcriptome sequencing and quantitative expression analysis

Transcriptome sequencing was performed at DNALINK, Inc. The purity and concentration of the RNA samples were measured with a NanoDrop™ 8,000 spectrophotometer (Thermo Fisher Scientific, Seoul, South Korea). Total RNA integrity was evaluated as an RNA Integrity Number (RIN) using a Bioanalyzer 2,100 system (Agilent, United States), and high quality RNA samples (RIN > 7) were used for the analysis. RNA samples were subjected to ribosomal RNA depletion using the Ribo-Zero Plus rRNA Depletion Kit for bacteria (Illumina), followed by cDNA library construction using TruSeq® RNA Sample Prep (Illumina) according to the manufacturer’s protocols. The resulting library was sequenced using a NovaSeq 6,000 System (Illumina). Raw reads were assembled and aligned with the reference AWRP genome using TopHat2 v2.0.13 with default parameters (Kim et al., 2013). Cufflinks v2.2.0 (Trapnell et al., 2010) was run with default parameters to calculate the fragments per kilobase of transcript per million (FPKM) values using the read data which were obtained with biological duplicate samples, and to identify differentially expressed genes (DEGs) between 46 T-a and AWRP for each phase. A differentially expressed gene was defined by the criteria of |log_2_(folds)| > 1 and false discovery rate (FDR) < 0.001.

## Results

### Adaptation of *Clostridium* sp. AWRP to acetate challenge

We first assessed the effect of acetate on the growth of wild-type AWRP on a mixture of the substrate gases CO_2_ (20%) and H_2_ (80%), as AWRP produces acetate as the major metabolite when using CO_2_ + H_2_ (see Figure 1 for details). In the absence of exogenous acetate, AWRP cells consumed about 90% of CO_2_ in the headspace within 72 h. However, the culture times for 90% CO_2_ consumption in the presence of 5 and 10 g L^−1^ (represented as the free acetic acid concentration throughout the paper) of added acetate were 1.5- and 3-fold longer than the non-supplemented cultures.

As the concentration of exogenous acetate increased, wild-type AWRP cells showed an increase in the lag of CO_2_ consumption despite the cells being able to grow in the presence of 10 g L^−1^ acetate (Figure 1A). Although AWRP cells consumed CO_2_ almost completely after a lag period of 4 days in terms of CO_2_ consumption, the lag period was not shortened after several serial passages in the presence of 5 g L^−1^ acetate (Supplementary Figure S1A), and the initial CO_2_ consumption rates did not exceed 10 mmol (L culture)^−1^ d^−1^ prior to the 10^th^ transfer (designated as 10 T; Supplementary Figure S2B). As serial transfer continued, a gradual increase in the CO_2_ consumption rate was observed from 15 T; the initial CO_2_ consumption rates were 28 and 40 mmol (L culture)^−1^ d^−1^ at 15 T and 45 T, respectively (Supplementary Figure S2B). Finally, cells from 46 T culture were spread on solid medium after serial dilutions, and eight colonies were randomly selected to investigate whether single clones still retain the phenotypic changes obtained during adaptive evolution. In the presence of 5 g L^−1^ exogenous acetate, all of the clones tested and the evolved population displayed similar CO_2_ consumption and final metabolite profiles (data not shown). Thus, one clone (referred to as 46 T-a) was chosen and subjected to the further characterization.

Compared to wild-type AWRP, 46 T-a showed shorter lag periods in gas consumption with acetate supplementation (Figure 1). In the absence of exogenous acetate, 46 T-a consumed CO_2_ at a rate similar to AWRP (Figure 1A). Interestingly, the final cell densities obtained with 46 T-a were more than twice of those obtained with AWRP (Figure 1B), even though total amounts of CO_2_ consumption were not different (Figure 1A). Furthermore, 46 T-a produced more ethanol than AWRP in all conditions tested. In the presence of 5 g L^−1^ acetate, 46 T-a almost exclusively produced ethanol (Figure 1B). Nevertheless, no significant difference was observed in the carbon and electron balances of AWRP and 46 T-a (Supplementary Table S2).

### Characterization of strains AWRP and 46 T-a without exogenous acetate

In small batch cultures, strains AWRP and 46 T-a produced only small amounts of metabolites due to the limited amount of gaseous substrates. In particular, the final concentration of acetate produced in both strains was 2 g L^−1^ or less (see Figure 1B). To determine whether the increased acetate tolerance of 46 T-a would enhance growth and metabolite production in the absence of acetate supplementation, growth, gas consumption, and metabolite production of AWRP and 46 T-a were determined with a larger culture volume (100 mL) with gas recharge. The results indicated that 46 T-a showed better growth and metabolite production than AWRP (Supplementary Figure S2). 46 T-a showed higher biomass yield than AWRP (Supplementary Figure S2A). AWRP no longer consumed CO_2_ after gas recharging at 60 h, whereas 46 T-a continued to consume gas after recharging at 48 h, producing more acetate than AWRP (3.4 vs. 2.5 g L^−1^; Supplementary Figure S2C). However, there was little difference in the final ethanol titers obtained with two strains (Supplementary Figure S2C).

**Figure 2:**
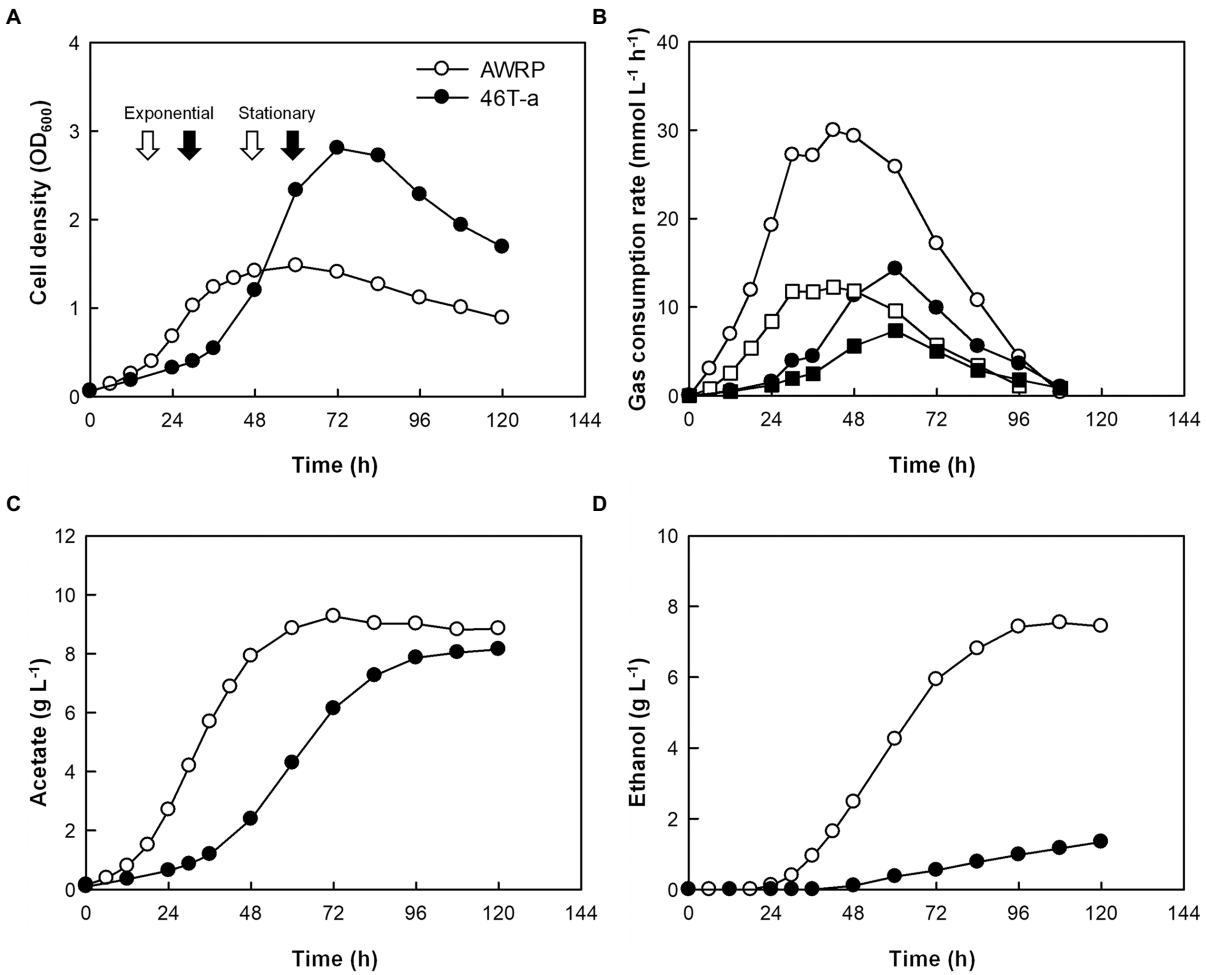
Batch fermentation profiles of AWRP (open symbols) and 46 T-a (closed symbols) without exogenous acetate. All fermentations were conducted in duplicate with continuous gas feeding and pH control. Average values of cell growth **(A)**, gas consumption rate **(B)**, acetate **(C),** and ethanol **(D)** production are shown. Samples for transcriptome analysis were taken at time points indicated by arrows in panel **A**. The squares and circles in panel **B** indicate the CO_2_ and H_2_ consumption rates, respectively. See Supplementary Table S3 for individual profiles.

The growth kinetics of AWRP and 46 T-a were compared using a bioreactor with pH controlled to 5.0 and a continuous gas feed (Figure 2; see Supplementary Figure S3 for individual profiles). The concentration of yeast extract was increased to 2 g L^−1^ to replenish more organic carbon sources for batch culture. As in the bottle cultures, the adapted strain grew slowly during its initial growth stage (Figure 2A), where the culture pH was higher than 5.0 (data not shown), but the maximum cell density of 46 T-a was almost twice (OD_600_ = 2.8) that of AWRP. This result is not striking in that 46 T-a was adapted near pH 5.0 (see Materials and Methods), and the optimum pH of the wild-type AWRP was found to be between 6.0 and 6.5 (Lee et al., 2019). It is intriguing that 46 T-a achieved higher cell density despite its lower gas consumption rate: specific gas consumption rates of 46 T-a were 17 and 35 mmol (g dry cell mass)^−1^ h^−1^ for CO_2_ and H_2_, respectively, each of which was about one-third of that the wild-type strain (Figure 2B). As a result, 46 T-a produced less metabolites, 8.1 and 1.3 g L^−1^ of acetate and ethanol, while AWRP produced 8.4 and 7.4 g L^−1^, respectively (Figures 2C,D).

**Figure 3:**
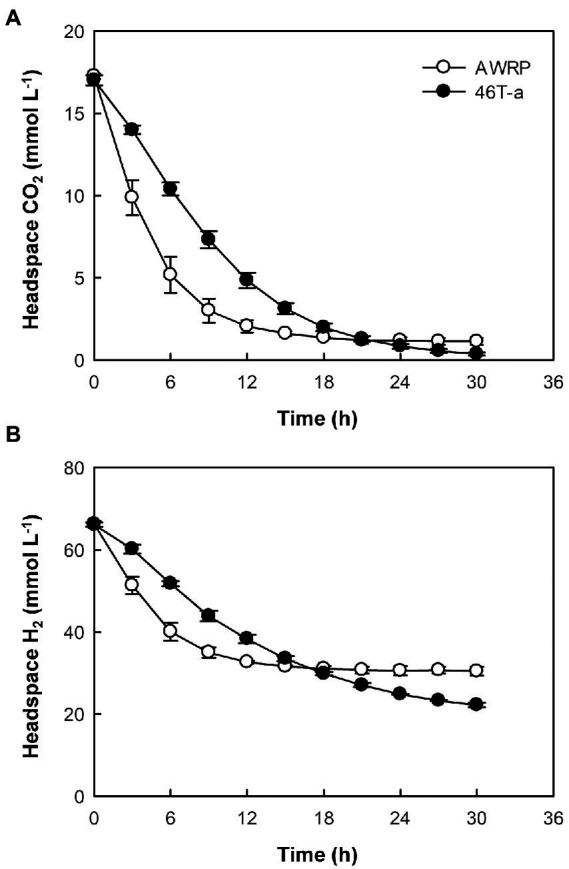
Time-course profiles of headspace CO_2_ **(A)** and H_2_ **(B)** in resting cell assay using cells of strains AWRP (open symbols) and 46 T-a (closed symbols). For each strain, assays were conducted in triplicate using medium without yeast extract and average values are presented. Error bars represent one standard deviation.

The low gas consumption and metabolite production of 46 T-a in the bioreactor experiment were found to be inconsistent with bottle cultivation result. To determine whether the decrease in gas consumption rate was due to an increase in yeast extract concentration, gas consumption rate was monitored by resting cell assay using a defined medium not supplemented with yeast extract (Figure 3). The gas consumption rates of 46 T-a were found to be lower than those of AWRP. The maximum specific gas consumption rates of 46 T-a were 19.2 and 41.8 mmol (g DCW)^−1^ h^−1^ for H_2_ and CO_2_, respectively, which were only half of those of AWRP. Interestingly, 46 T-a cells continued to consume H_2_ at low levels of CO_2_, while AWRP cells began to decline sharply when the headspace CO_2_ concentration was less than 5 mmol L^−1^ (Figure 3B).

### Genome sequencing of strain 46 T-a and identification of mutations

The genome of 46 T-a was sequenced to identify phenotypic-associated mutations, and four mutated genes were identified in the genome (Table 1). All these mutations were also identified in other clones and in the 46 T population by Sanger sequencing (data not shown). Interestingly, three of these mutations were located in a gene cluster encoding an electron-bifurcating hydrogenase complex (Hyt), which is known to play a pivotal role in utilizing H_2_ and reducing CO_2_ to formate (Wang et al., 2013; Mock et al., 2015): *hytB* (DMR38_03370; encoding flavin mononucleotide protein), *hytD* (DMR38_03375; iron–sulfur protein), and *hytE1* (DMR38_03380; iron–sulfur protein), all of which are likely to be involved in electron transfer during enzyme reaction. The mutation in *hytD*, which replaced threonine with isoleucine, is within the [4Fe-4S] ferredoxin-like domain (Table 1). In addition, the mutation found in the *hytB* gene is close to the soluble ligand binding domain (SLBB; residues 356^th^ to 404^th^ of HytB) containing a conserved motif present in electron-bifurcating enzymes (Losey et al., 2020).

**Table 1:** Mutations identified in the 46 T-a genome.

Locus tag	Annotation	Mutation	Amino acid change	Predicted domain in which the mutation is located
DMR38_03370	NADP^+^-dependent electron-bifurcating hydrogenase subunit B	G1261T	V421F	None
DMR38_03375	NADP^+^-dependent electron-bifurcating hydrogenase subunit D	C422T	T141I	[4Fe-4S] ferredoxin-type, iron–sulfur binding domain (IPR017896)
DMR38_03380	NADP^+^-dependent electron-bifurcating hydrogenase subunit E1	C365T	T122I	None
DMR38_03800	Molecular chaperone DnaK	G67T	D23Y	ATPase, nucleotide binding domain (IPR043129)

In addition to mutations in the genes encoding Hyt complex, the 46 T-a genome harbors a single point mutation (G67T) in the *dnaK* gene, which encodes a Class I molecular chaperone. In other clostridial species, acid stresses has been reported to result in up-regulation of *dnaK* and other heat shock proteins (HSPs; Alsaker et al., 2010; Suo et al., 2017). This mutation results in the substitution of aspartate at position 23^rd^ with a tyrosine in DnaK (Table 1). Based on the structure of the *E*. *coli* DnaK protein, the residue is located within the putative N-terminal ATPase domain, but the 23^rd^ residue does not appear to be near the ATP-binding pocket (Qi et al., 2013).

### Transcriptional changes in the acetate-adapted strain

In addition to the whole genome sequencing of 46 T-a, comparative transcriptome analysis between AWRP and 46 T-a was performed using samples from bioreactor experiments to investigate how the transcriptome profile of 46 T-a would change from that of AWRP. Samples were collected at exponential and stationary phases in duplicate fermentations of each strain (see Figure 2 for sampling points for transcriptome profiling). High-quality reads were mapped onto the AWRP genome with mapping rates over 95% in all of the samples (Supplementary Table S3), and all the correlation coefficients between two biological replicates were more than 0.98 (data not shown). Differentially expressed gene (DEG) analysis indicated that 46 T-a showed global changes in the transcriptome despite several point mutations occurring in the genome: a total of 601 and 740 genes were found to be differentially expressed (|log_2_[fold-change]| > 1 and false discovery rate [FDR] < 0.001) at the exponential and stationary phase, respectively (see Supplementary Tables S4, S5 for entire transcriptome profiles at two phases). KEGG Orthology analysis of DEGs showed that the adaptation process led to down-regulation of many metabolic genes (Category 1 to 11 in Figure 3): 149 and 172 genes were down-regulated with statistical significance (FDR < 0.001), whereas only 40 and 79 genes were up-regulated at exponential and stationary phases, respectively. At the exponential phase, the proportion of DEGs was highest in Amino acid metabolism (23%; Category 5 in Figure 3), followed by Nucleotide metabolism (21%; Category 4), and Folding, sorting and degradation (20%; Category 14). At the stationary phase, Amino acid metabolism still displayed the highest proportion of DEGs (38%), followed by Translation (34%; Category 13), and Nucleotide metabolism (33%), all of which are closely related to cell growth.

**Figure 4:**
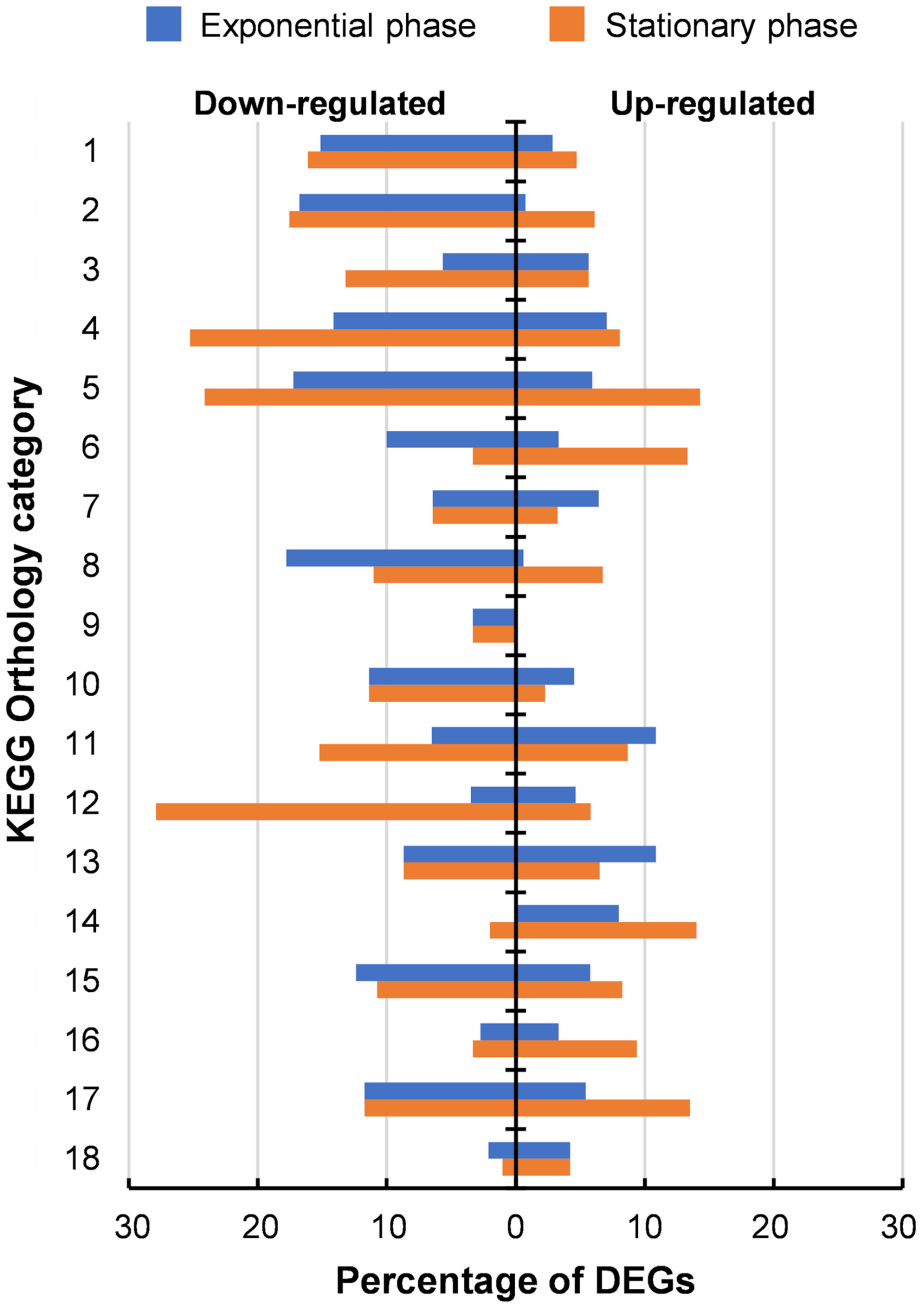
Distribution of differentially expressed genes (DEGs) identified in 46 T-a in comparison with AWRP. DEGs are categorized based on KEGG pathway annotations. The numbered categories are: 1, carbohydrate metabolism (211); 2, energy metabolism (131); 3, lipid metabolism (53); 4, nucleotide metabolism (99); 5, amino acid metabolism (203); 6, metabolism of other amino acids (30); 7, glycan biosynthesis and metabolism (31); 8, metabolism of cofactors and vitamins (163); 9, metabolism of terpenoids and polyketides (30); 10, biosynthesis of other secondary metabolites (44); 11, xenobiotics biodegradation and metabolism (46); 12, translation (86); 13, folding, sorting and degradation (46); 14, replication and repair (50); 15, membrane transport (121); 16, signal transduction (181); 17, cellular community (111); and 18, cell motility (95). The number in parentheses after each category is the total number of annotated genes in the AWRP genome.

**Figure 5:**
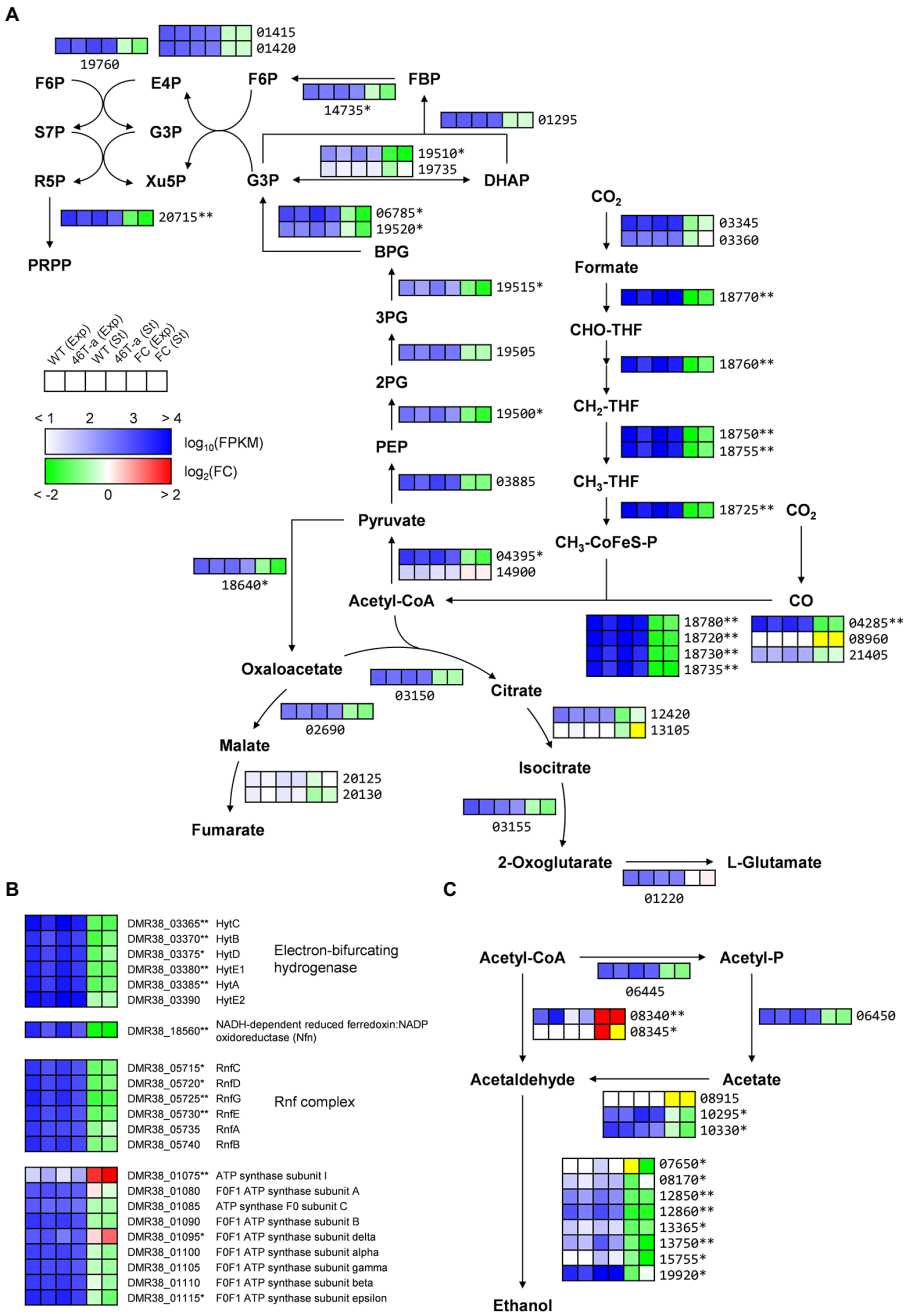
Transcription profiles of central metabolic pathways in AWRP and 46 T-a. **(A)** Shown are central carbon metabolism including the Wood-Ljungdahl pathway, glycolysis/gluconeogenesis, pentose phosphate pathway, and bifurcating TCA cycle. **(B)** Shown are genes involved in electron transfer and ATP synthesis. **(C)** Shown are genes involved in acetate and ethanol production. Transcription levels (represented as Fragments Per Kilobase Million [FPKM]) and fold changes for each gene are shown as a six-column heatmap. From left to right, the first 4 boxes show the log_10_(FPKM) value of AWRP in exponential phase (Exp), 46 T-a (Exp), AWRP in stationary phase (St), and 46 T-a (St). The right two boxes show the fold change (FC) of exponential and stationary phase expressed as log_2_(FPKM_46T-a_/FPKM_AWRP_). The yellow color in the fold change box indicates that the fold change value might not be valid because both FPKM values were too low (<10). The gene’s locus tag is located to the right or below the heatmap box (prefixes are omitted in panels **A**,**C** for simplicity). Single and double asterisks next to the locus tag indicate differential expression with statistical significance (|log_2_(folds)| > 1 and FDR < 0.001) in one and two phases, respectively.

Many of the genes that were most up-regulated or down-regulated in 46 T-a were poorly annotated genes (Supplementary Tables S4, S5). Nevertheless, differential expression of the metabolic genes that might contribute to the observed phenotype of the adapted strain was identified. Interestingly, most genes involved in carbon fixation were significantly down-regulated in adapted strain from both phases (Figures 4, 5). Most genes involved in the WL pathway are encoded in a large gene cluster (DMR38_18715–80; *ca*. 16 kb), all of which were 2- to 4-fold down-regulated in 46 T-a from both phases (Figure 5A; Supplementary Tables S4, S5). The formate-dehydrogenase-encoding genes (DMR38_03345 for FdhA and 03360 for FdhD) were not significantly changed (Figure 5A). Most genes involved in glycolysis/gluconeogenesis, the pentose phosphate pathway, and the incomplete TCA cycle (Figure 5A) were found to be down-regulated. Other auxiliary enzymes for energy conservation including the Hyt hydrogenase, the NADH-dependent ferredoxin:NADP^+^ oxidoreductase (Nfn), and the Rnf complex, all of which are essential for autotrophic growth in CO_2_ + H_2_ (Tremblay et al., 2013; Woods et al., 2022), were also significantly down-regulated in 46 T-a (Figure 5B). Regarding ethanol production, two of three genes encoding aldehyde:ferredoxin oxidoreductase (AOR; DMR38_10295 and DMR38_10330), which were actively transcribed in AWRP, were significantly down-regulated in 46 T-a at the stationary phase (Figure 5C). Most genes encoding orphan alcohol dehydrogenase were also down-regulated, but *adhE1* gene (DMR38_08340; encoding bifunctional aldehyde/alcohol dehydrogenase) was significantly up-regulated in 46 T-a from both phases (Figure 5C).

**Figure 6:**
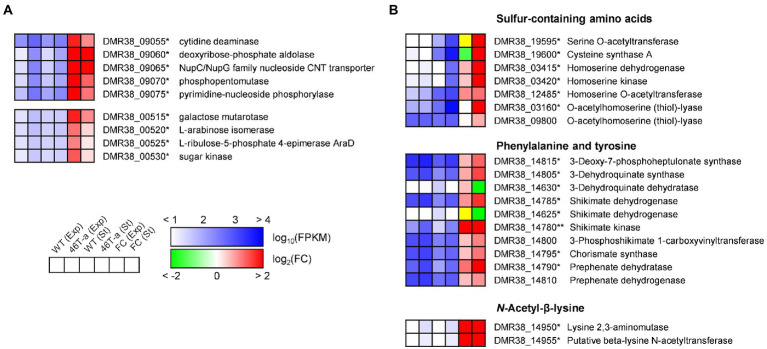
Transcription profiles of selected genes involved in carbohydrate utilization **(A)** and amino acid biosynthesis **(B)**. The colorimetric scale is the same as in Figure 5. The yellow color in the fold change box indicates that the fold change value might not be valid because both FPKM values were too low (<10). Single and double asterisks next to the locus tag indicate differential expression with statistical significance (|log_2_(folds)| > 1 and FDR < 0.001) in one and two phases, respectively.

While many DEGs involved in central metabolism and anabolic activity were down-regulated, several genes involved in carbohydrate utilization and amino acid biosynthesis were up-regulated in 46 T-a (Figure 6). One gene cluster (DMR38_09055–75) contains the genes required for nucleoside uptake and degradation of pyrimidine deoxyribonucleosides into glyceraldehyde-3-phosphate (G3P), and acetaldehyde (Lees and Jago, 1977; Figure 6A). A putative arabinoside degradation operon (DMR38_00515–30) consisting of genes encoding a sugar kinase, an L-ribulose-5-phosphate 4-epimerase, an arabinose isomerase, and a galactose mutarotase was also significantly up-regulated in 46 T-a (Figure 6A). 46 T-a was also found to exhibit strong up-regulation of genes involved in serine (DMR38_00400, 00405, and 00415), cysteine (DMR38_19595 and 19600), and methionine biosynthesis (DMR38_03415, 03420, 12485, 03155, 03160, and 09800) as well as the methionine-transporter-encoding gene (DMR38_16080) (Figure 6B). The biosynthesis pathways of aromatic amino acids except tryptophan were also up-regulated in 46 T-a. In addition, a putative operon for the biosynthesis of *N*^ε^-acetyl-β-lysine (ABL), known as an osmoprotectant in some archaea species (Sowers et al., 1990), was up-regulated in 46 T-a (DMR38_14950 and 14955; Figure 6B).

**Figure 7:**
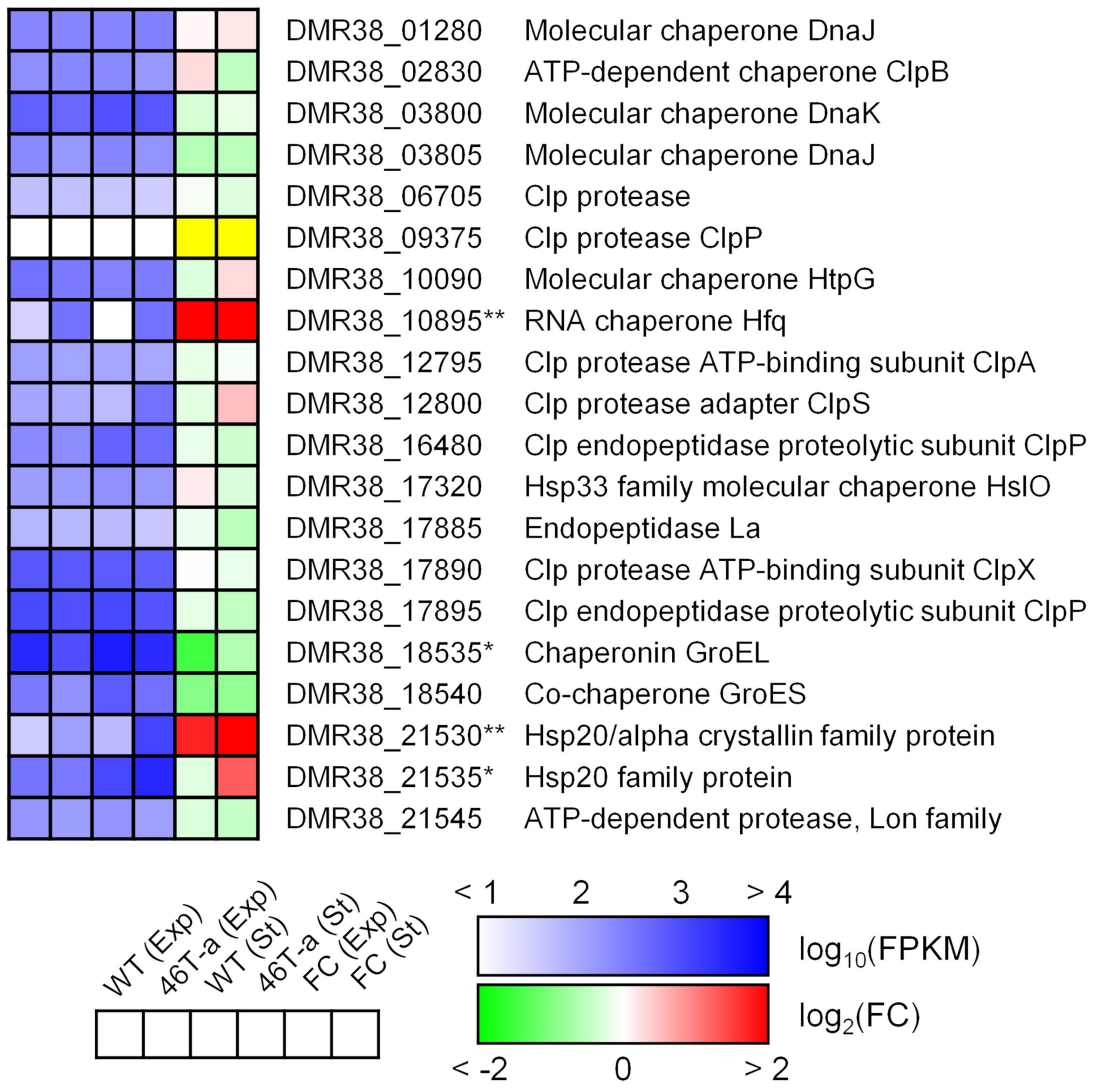
Transcription profiles of selected genes involved in general stress response. The colorimetric scale is the same as in Figure 5. The yellow color in the fold change box indicates that the fold change value might not be valid because both FPKM values were too low (<10). Single and double asterisks next to the locus tag indicate differential expression with statistical significance (|log_2_(folds)| > 1 and FDR < 0.001) in one and two phases, respectively.

Stress shock, including acetate challenge, is known to induce up-regulation of various heat shock genes in the model Clostridial species *C*. *acetobutylicum* (Bahl et al., 1995; Tomas et al., 2003; Yoo et al., 2020). Our transcriptome results indicated that only a few of those genes were up-regulated in the acetate-adapted strain; only genes encoding Hsp20 family proteins were up-regulated (DMR38_21530 and 21535; Figure 7). Interestingly, *hfq* (DMR38_10895; encoding RNA chaperone) was significantly up-regulated in 46 T-a (Figure 7). Hfq is known to interact with various small RNAs present in bacteria to control target genes complementary to small RNAs (Kavita et al., 2018).

### Growth of 46 T-a without CO_2_ + H_2_

The results from the comparative transcriptome analysis between 46 T-a and AWRP and the resting cell assay indicated that the expression levels of the central carbon metabolism, which was consistent with the fermentation kinetics of 46 T-a. However, the mutations and the transcriptome changes were not in agreement with the possibility that the adapted strain was able to synthesize biomass from CO_2_ + H_2_ more efficiently than the wild-type strain (see Figure 4). Therefore, we determined whether the adapted strain was able to grow only using the organic ingredients supplemented to the medium during ALE (yeast extract, methionine, and cysteine). Indeed, 46 T-a showed weak growth in culture medium even in the absence of gaseous substrates, whereas the wild-type strain was unable to grow under the same condition (Supplementary Figure S4).

## Discussion

In this study, we observed that AWRP showed an extended lag phase in gas consumption during growth in CO_2_ + H_2_ when culture medium was provided with a moderate concentration of acetate (5 or 10 g L^−1^) (Figure 1). ALE experiment showed that the lag phase due to acetate supplementation was shortened after 46 transfers in the presence of 5 g L^−1^ acetate (Supplementary Figure S1), and the adapted strain 46 T-a consumed CO_2_ more rapidly than AWRP, especially in the presence of exogenous acetate (Figure 1A). Even with no acetate supplementation, 46 T-a showed more gas consumption even though there was almost no difference in the specific growth rate of 46 T-a and AWRP (Supplementary Figure S2).

In *C*. *acetobutylicum*, the model species of the genus *Clostridium*, it has been demonstrated that metabolite challenge led to significant up-regulation of HSP-encoding genes, including *dnaK* (Tomas et al., 2003; Alsaker et al., 2010; Venkataramanan et al., 2013). The expression level of *dnaK* did not change significantly in 46 T-a (Figure 7). Instead, we observed strong up-regulation of *hfq* (Figure 7). It is noteworthy that there is increasing evidence for the link between DnaK and Hfq (Guisbert et al., 2007; Lei et al., 2015). Thus, one possible hypothesis is that mutation in the *dnaK* gene may affect the behavior of Hfq expression as up-regulated in 46 T-a. Many of small RNA (sRNA) genes induced upon metabolite challenge in *C*. *acetobutylicum* have Hfq-binding motifs, revealing a role for Hfq in the stress response (Venkataramanan et al., 2013). Although 25 sRNA genes have been identified in *C*. *ljungdahlii*, their roles are still unknown (Tan et al., 2013). Further investigation is needed to address the effects of *dnaK* gene mutation and the relationship between up-regulation of *hfq* and expression of sRNA genes, which will be useful for understanding metabolite stress responses in industrially important acetogens.

Several amino acid biosynthesis pathways were up-regulated in 46 T-a (Figure 6B). In the stationary phase (corresponds to approximately 6 g L^−1^ acetate; see Figures 2A,C), 46 T-a showed strong up-regulation of the genes involved in serine, cysteine, and methionine biosynthesis (Figure 6B). Previous studies have shown that biosynthesis of these amino acids was inhibited by acetate challenge, and supplementation with these amino acids restored growth retardation (Roe et al., 2002; Alsaker et al., 2010). Furthermore, up-regulation of biosynthesis pathways was observed for chorismate, phenylalanine, tyrosine, but not tryptophan (Figure 6B), which was observed in *C*. *acetobutylicum* upon butyrate or butanol stress (Alsaker et al., 2010). These results are in consistent with the previous studies that certain amino acids could relieve metabolite stress. Interestingly, we observed in 46 T-a up-regulation of the genes involved in ABL biosynthesis (Figure 6B); orthologous genes were not identified by BLAST searches in the genomes in *C*. *ljungdahlii* and *C*. *autoethanogenum* (data not shown). The role for ABL has been studied in archaea, but there is also evidence that these genes are induced upon salt stress in bacterial species (Triado-Margarit et al., 2011). Increased ABL biosynthesis is likely to favor the growth of 46 T-a in the presence of exogenous acetate. Bacterial cells can also mitigate acetate stress by modifying membrane composition (Trček et al., 2007). For example, it has been shown that acetate challenge induced *C*. *acetobutylicum* to up-regulate the *cfa* gene, which encodes a cyclopropane fatty acid synthase (Alsaker et al., 2010). No differential expression of the relevant genes was observed in this study, suggesting that membrane composition might not be a key factor for higher acetate tolerance in 46 T-a. Acetate tolerance can be increased by up-regulation of the acetate transporter, but it could not be determined in this study because the AWRP genome does not encode known orthologs of the acetate transporter. Based on the prediction from thermodynamic metabolic flux analysis in *C*. *autoethanogenum*, it has been suggested that intracellular acetate would be exported through the uniporter of acetate anions, of which the genetic identity has not been identified (Mahamkali et al., 2020).

The previous study observed that the enzymes for ethanol production were always strongly expressed in *C*. *ljungdahlii* and proposed an overflow model for ethanol synthesis, in which accumulation of intracellular acetate and reduced ferredoxin are prerequisites for the conversion of acetate to acetaldehyde *via* the AOR enzyme (Richter et al., 2016). In *C*. *autoethanogenum*, it has been suggested that the accumulation of extracellular acetate accompanied by high biomass increased the uncoupling of proton motive force, thereby increasing the maintenance cost of cells (Valgepea et al., 2017a; Mahamkali et al., 2020). To meet the ATP demand, metabolism shifted from carbon fixation to CO oxidation to provide more reduced ferredoxin used to convert acetate to ethanol *via* AOR and alcohol dehydrogenase (ADH; Valgepea et al., 2017a). Consistent with these hypotheses, wild-type AWRP produced more ethanol in the presence of 5 g L^−1^ acetate (Figure 1B). However, providing more acetate (10 g L^−1^) completely diminished ethanol production by AWRP. Although the reason was not determined in this study, the results indicate that somehow availability of reduced ferredoxin was not sufficient to drive the AOR reaction in AWRP with 10 g L^−1^ of exogenous acetate. Interestingly, 46 T-a produced more ethanol than AWRP in small-scale culture, and net acetate production was much lower especially when exogenous acetate was provided (Figure 1B). Such a high ethanol yield from CO_2_ + H_2_ has not been observed in the other alcohol-producing acetogens (Mock et al., 2015; Zhu et al., 2020; Liew et al., 2022). This may be due to the different behavior of H_2_ consumption in strains as observed in resting cell assay (Figure 3). In AWRP, H_2_ consumption rates decreased dramatically as the headspace CO_2_ level was less than 5 mmol L^−1^. On the other hand, 46 T-a showed lower H_2_ consumption rates at the beginning of the analysis, but H_2_ consumption continued at low CO_2_ level, resulting in more H_2_ consumption (Figure 3). The results suggest that ethanol production from acetate in 46 T-a might occur when relative availability of CO_2_ was low, which also explains the results of large-scale cultures, supplied with additional CO_2_ by gas recharge or continuous blowing (Supplementary Figure S2). Further studies are necessary for identification of key mutations (i.e., related to the different H_2_ consumption behavior in 46 T-a) among those identified in this study. In bioreactor experiments, *adhE1* was strongly up-regulated in 46 T-a (Figure 5C), but ethanol production *via* the acetaldehyde/alcohol dehydrogenase pathway (i.e., direct reduction of acetyl-CoA to acetaldehyde; see Figure 5C) was shown to lead to negative ATP gain in CO_2_ + H_2_ (Bertsch and Müller, 2015; Mock et al., 2015); at this time, the benefits are unclear.

ALE actually increased the fitness of 46 T-a in the presence of acetate, and the strain exhibited higher cell densities even without acetate supplementation. One question is how this strain was able to produce a greater amount of cells despite specific gas consumption rates. Obviously, the strain showed lower gas consumption rates than AWRP (see Figures 2, 3). Because the ATP yield obtained through the WL pathway is determined by thermodynamics and stoichiometry (Schuchmann and Müller, 2014), it is almost impossible to increase the ATP yield of 46 T-a as a result of ALE. The remaining possibility is that 46 T-a has been adapted to reduce maintenance costs that can be achieved by (i) utilization of organic compounds and (ii) reduction of unnecessary metabolic expenditure through re-allocation of cellular resources. It has been reported that some acetogens can utilize amino acid metabolism during autotrophic growth, which replenishes additional ATP to promote growth (Aklujkar et al., 2017; Valgepea et al., 2017b). For example, previous studies on *C*. *ljungdahlii* and *C*. *autoethanogenum* suggested that degradation of L-arginine and L-ornithine could provide additional ATP acquisition and promote cell growth under autotrophic conditions (Aklujkar et al., 2017; Valgepea et al., 2017b). The arginine deiminase (ADI; encoded by DMR38_04360), however, was rarely expressed in both AWRP and 46 T-a strains and was not significantly up-regulated in 46 T-a (Supplementary Tables S3, S4). In addition, the ornithine degradation pathway was significantly down-regulated in 46 T-a. All these results indicate that neither pathway contributed to the enhanced growth of 46 T-a. Instead, significant up-regulation of nucleoside degradation and arabinoside metabolism was observed (Figure 6A), which would help the cells to uptake and utilize exogenous nutrients supplemented as yeast extract. Obviously, these pathways might not be the main pathways for energy production, as the growth of 46 T-a on yeast extract was insignificant in the absence of CO_2_ + H_2_ (Supplementary Figure S4). This is possibly because the AWRP genome does not encode the degradation pathways for amino acids and nucleotides present in aminolytic and purinolytic clostridia (Fonknechten et al., 2010; Hartwich et al., 2012). Nevertheless, the dissimilatory metabolism up-regulated in 46 T-a might reduce ATP consumption for the biosynthesis of building blocks or provide additional ATP through glycolysis and pentose pathway that compensate for reduced carbon fixation. In this study, we observed up-regulation of the nucleoside transporter (Figure 6A) and the methionine transporter (DMR38_16080; see Supplementary Table S5) in 46 T-a. In addition, a couple of transporter genes were found to be up-regulated in this strain (Supplementary Tables S4, S5). Because their substrate is unclear, further work is needed to confirm the hypothesis.

Contrary to our expectations, the enhanced acetate tolerance of 46 T-a did not lead to more acetate production in the bioreactor experiments (Figure 2). As discussed above, our study indicates that acetogen has the potential to favor alternative substrates for growth over gas during long-term cultivation. This is undesirable for practical applications. Nevertheless, the physiological changes in 46 T-a, such as a modified ethanol metabolism, is worth investigating in the future to gain insight into strategies for efficient alcohol production from CO_2_ + H_2_. Furthermore, understanding the rationale for the reduced gas consumption can have practical implications for conducting continuous CO_2_ fermentation (Acharya et al., 2019; Liew et al., 2022).

## Data availability statement

The datasets presented in this study can be found in online repositories. The names of the repository/repositories and accession number(s) can be found in the article/Supplementary material.

## Author contributions

SK performed the cultivation experiments, data analysis, and manuscript writing as a main researcher. JL performed RNA isolation and transcriptome analysis. JL and HL participated in experimental design, conception, funding, and manuscript writing. All authors contributed to the article and approved the submitted version.

## Funding

This work was financially supported by the KIOST In-House Program (grant number PEA0022).

## Conflict of interest

The authors declare that the research was conducted in the absence of any commercial or financial relationships that could be construed as a potential conflict of interest.

## Publisher’s note

All claims expressed in this article are solely those of the authors and do not necessarily represent those of their affiliated organizations, or those of the publisher, the editors and the reviewers. Any product that may be evaluated in this article, or claim that may be made by its manufacturer, is not guaranteed or endorsed by the publisher.
