# Supplementary material for: Metabolic changes of the acetogen Clostridium sp. AWRP through adaptation to acetate challenge
Source: Front Microbiol. 2022 Dec 7;13:982442. doi: 10.3389/fmicb.2022.982442 (PMC9768041; doi:10.3389/fmicb.2022.982442)
Supplement: Supplementary file 1 [file Data_Sheet_1.DOCX]

Supplementary Material

# Supplementary Figures and Tables

## Supplementary Figures

**Figure S1.** Adaptation of *Clostridium* sp. AWRP to acetate challenge with CO_2_ plus H_2_ as the autotrophic substrate. (**A**) Time-course CO_2_ consumption profile of AWRP in the early stages of adaptation in the presence of 5 g L^-1^ acetate. (**B**) Initial CO_2_ consumption rates during the adaptation to the acetate challenge.

**Figure S2.** Time-course profiles of cell growth (**A**), CO_2_ consumption (**B**), and metabolite production (**C**) from the cultures of the wild-type AWRP (closed symbols) and 46T-a (open symbols), which were carried out with gas recharge. Acetate was not supplemented. The time at which gas was recharged was indicated arrows in **A**. Cultures were performed in triplicate for each strain, and average values are shown. Error bars indicate one standard deviation.

**Figure S3.** Individual profiles of AWRP (**A**) and 46T-a (**B**) of bioreactor experiments in Fig. 3.

**Figure S4.** Growth of AWRP and 46T-a on 2 g L^-1^ yeast extract in the absence of CO_2_ + H_2_ (*i.e.*, grown under 100% N_2_ atmosphere). Shown are the final OD values measured after 4-day cultivation, and starting OD was ~ 0.05 for both strains.

## Supplementary Tables

**Table S1**. Primers used in this study

| Name | Sequence | Description |
| --- | --- | --- |
| 03370_F | TATGGTAGGAGAACCTAGAG | For amplification of *hytB* (DMR38_03370); 03370_F was used for Sanger sequencing |
| 03370_R | TTACTGGATTTGGAGCACTC |  |
| 03375_F | CAGTGATGCATTGCCTGGGC | For amplification of *hytD* (DMR38_03375); 03375_F was used for Sanger sequencing |
| 03375_R | TGGCTGCTCTTTTCCAAGTC |  |
| 03380_F | GTGAGATTGCCTGTGCTGCA | For amplification of *hytE1* (DMR38_03380); 03380_F was used for Sanger sequencing |
| 03380_R | CCGCACATCGTCTACAGCCT |  |
| 03800_F | GGAAATTCCTTGCGAAGGAG | For amplification of *dnaK* (DMR38_03800); 03800_R was used for Sanger sequencing |
| 03800_R | AATCCTGCAATCTTACCTGC |  |

**Table S2.** Carbon and electron balances of AWRP and 46T-a using gas (CO_2_ and H_2_) consumption, metabolite (acetate and ethanol) production, and biomass formation in small-scale batch cultures.

|  | AWRP | | | | | | | 46T-a | | | | | | |  |
| --- | --- | --- | --- | --- | --- | --- | --- | --- | --- | --- | --- | --- | --- | --- | --- |
| Acetate provided  (g L^-1^) | CO_2_ consumption (mmol) | H_2_ consumption (mmol) | Acetate (mmol) | Ethanol (mmol) | Biomass  (μmol) | Carbon recovery (%) | Electron balance (%) | CO_2_ consumption (mmol) | H_2_ consumption (mmol) | Acetate (mmol) | Ethanol (mmol) | Biomass  (μmol) | Carbon recovery (%) | Electron balance (%) | |
| 0 | 1.8 ± 0.3 | 4.9 ± 0.9 | 0.7 ± 0.0 | 0.3 ± 0.1 | 77.6 ± 20.2 | 108.3 ± 3.1 | 89.9 ± 3.9 | 2.1 ± 0.0 | 6.2 ± 0.1 | 0.3 ± 0.0 | 0.6 ± 0.0 | 239.9 ± 17.2 | 101.3 ± 0.8 | 80.3 ± 0.8 | |
| 5 | 2.0 ± 0.0 | 5.6 ± 0.3 | 0.5 ± 0.1 | 0.5 ± 0.1 | 63.9 ± 4.0 | 101.3 ± 2.0 | 85.7 ± 1.1 | 2.0 ± 0.0 | 6.4 ± 0.1 | NA^a^ | 0.9 ± 0.0 | 214.6 ± 4.8 | 101.9 ± 2.3 | 86.3 ± 0.9 | |
| 10 | 1.8 ± 0.0 | 4.2 ± 0.1 | 0.9 ± 0.0 | NA^a^ | 42.2 ± 2.2 | 99.6 ± 4.2 | 84.5 ± 2.8 | 1.9 ± 0.0 | 5.9 ± 0.3 | 0.1 ± 0.1 | 0.8 ± 0.1 | 161.8 ± 8.9 | 100.5 ± 3.6 | 84.9 ± 2.0 | |

^a^NA, not available.

**Table S3**. Brief statistics of the RNA-seq experiments

| Strain | Phase | Run | Reads | Mapped reads | Mapping rate (%) |
| --- | --- | --- | --- | --- | --- |
| AWRP | Exponential | 1 | 47,733,214 | 46,466,589 | 97.3 |
|  |  | 2 | 45,700,310 | 44,436,486 | 97.2 |
|  | Stationary | 1 | 45,644,128 | 44,588,595 | 97.7 |
|  |  | 2 | 48,781,668 | 47,125,818 | 96.6 |
| 46T-a | Exponential | 1 | 52,504,310 | 51,334,350 | 97.8 |
|  |  | 2 | 60,034,338 | 57,926,513 | 96.5 |
|  | Stationary | 1 | 50,392,332 | 48,287,464 | 95.8 |
|  |  | 2 | 56,451,276 | 54,679,859 | 96.9 |
